# Microarchitecture of Heterotopic Ossification in Fibrodysplasia Ossificans Progressiva: An HR-pQCT Case Series

**DOI:** 10.3389/fcell.2021.627784

**Published:** 2021-03-11

**Authors:** Esmée Botman, Melissa S. A. M. Bevers, Caroline E. Wyers, Bert van Rietbergen, Bernd P. Teunissen, Pieter G. Raijmakers, Jan Coen Netelenbos, Joop P. van den Bergh, Elisabeth M. W. Eekhoff

**Affiliations:** ^1^Department of Internal Medicine Section Endocrinology, Amsterdam Bone Center, Amsterdam Movement Sciences, Amsterdam UMC, Vrije Universiteit Amsterdam, Amsterdam, Netherlands; ^2^Department of Internal Medicine, VieCuri Medical Center, Venlo, Netherlands; ^3^NUTRIM School for Nutrition and Translational Research in Metabolism, Maastricht University Medical Center, Maastricht, Netherlands; ^4^Orthopedic Biomechanics, Department of Biomedical Engineering, Eindhoven University of Technology, Eindhoven, Netherlands; ^5^Department of Internal Medicine, Subdivision Rheumatology, Maastricht University Medical Center, Maastricht, Netherlands; ^6^Department of Orthopedic Surgery, Maastricht University Medical Center, Maastricht, Netherlands; ^7^Department of Radiology and Nuclear Medicine, Vrije Universiteit Amsterdam, Amsterdam UMC, Amsterdam, Netherlands; ^8^Department of Medicine and Life Sciences, Hasselt University, Hasselt, Belgium

**Keywords:** fibrodysplasia ossificans progressiva, heterotopic ossification, high-resolution peripheral quantitative computed tomography, bone strength, bone microarchitecture

## Abstract

It is challenging to study heterotopic ossification (HO) in patients with fibrodysplasia ossificans progressiva (FOP) due to the contraindication of invasive techniques (*i.e.*, bone biopsies), which can trigger flare-ups. The aim of this case study was to assess mature HO at the microarchitectural level non-invasively with high-resolution peripheral quantitative computed tomography (HR-pQCT). Depending on the patient’s mobility, HR-pQCT scans were acquired of peripherally located HO and standard distal radius and tibia regions in two FOP patients, a 33-year-old woman and a 23-year-old man, with the classical mutation (p.R206H). HO was located around the halluces, the ankles, and in the Achilles tendon. Standard HR-pQCT analyses were performed of the distal radius, tibia, and HO to quantify bone mineral density (BMD) and bone microarchitecture. Micro-finite element analysis was used to estimate failure load (FL). The outcomes were compared between HO and neighboring skeletal bone and with an age- and gender-matched normative dataset from literature. The bone parameters of the radius were within the interquartile range (IQR) of normative data. In contrast, in the tibiae of both patients, total and trabecular BMD were below the IQR, as were trabecular bone volume fraction, number, and thickness, cortical thickness, and FL. Trabecular separation and heterogeneity were above the IQR. Isolated HO in the Achilles tendon had a lower total, trabecular, and cortical BMD, trabecular bone volume fraction, and cortical thickness than the normative tibia data. Trabecular microarchitecture was within the IQR, and FL was approximately 10% higher than that of the neighboring tibia after accounting for areal differences. Other scanned HO could only be qualitatively assessed, which revealed coalescence with the neighboring skeletal bone, development of a neo-cortex, and partial replacement of the original skeletal cortex with trabeculae. To conclude, isolated HO seemed microarchitecturally more comparable to reference tibia data than the peripheral skeleton of the FOP patients. HO and skeleton also appear to be able to become one entity when contiguous.

## Introduction

Fibrodysplasia ossificans progressiva (FOP) is a rare genetic disease that is characterized by the formation of heterotopic ossification (HO) in ligaments, tendons, and muscles (Rogers and Geho, 1979; Cohen et al., 1993; Kaplan et al., 2008). The formation of HO is often preceded by a clinical flare-up whose clinical signs are, among others, pain, redness, and swelling (Kaplan et al., 2008; Pignolo et al., 2016). The histology of these flare-ups developing into HO has previously been studied through biopsies that were obtained for other purposes, mainly to exclude malignancies in non-diagnosed FOP patients (Kaplan et al., 1993; Gannon et al., 1998). It is thought that, in the early stage of this HO development, the infiltration of inflammatory cells, such as lymphocytes, mast cells, and macrophages, causes cell death of the affected connective tissue. Proliferation of fibroblasts is thought to play a crucial role in the successive HO stage (Gannon et al., 2001). Finally, the fibroproliferative tissue develops into cartilage before it develops into endochondral bone (Kaplan et al., 1993; Gannon et al., 1998; Pignolo et al., 2011).

Less is known about mature HO. The few histological and radiological case reports suggest HO to follow a normal endochondral process, with deposition of bone matrix that visually appears indistinguishable from skeletal bone matrix with similar bone modeling and remodeling (Lutwak, 1964; Kaplan et al., 1994; Mahboubi et al., 2001). When mature, HO seems to consist of both compact and lamellar bone structures and apparently normal bone marrow (Jayasundara et al., 2012; Kamal et al., 2015). In FOP patients with the classic mutation (p.R206H), the skeletal bone is also assumed to develop normally despite developmental anomalies such as the frequently present shortened toes and the less frequently present short femoral neck and fusion of cervical facet joints (Kaplan et al., 2008). These case reports are mainly qualitative as a quantitative comparison of mature HO and skeletal bone remains difficult. Furthermore, histological examinations of mature human FOP HO are limited due to a contraindication of invasive techniques, such as bone biopsies, in FOP patients, as these techniques can trigger a flare-up and consequently aggravate the disease (Kitterman et al., 2005; Pignolo et al., 2016).

High-resolution peripheral quantitative computed tomography (HR-pQCT) may possibly alleviate the difficulty in investigating mature HO. This high-resolution imaging modality allows non-invasive and quantitative assessment of peripheral bones at the microarchitectural level, including quantification of geometry, density, and microarchitecture of the cortical and trabecular bone compartments and of the biomechanical properties of bone. Until now, HR-pQCT has mainly been used to study the distal radius and tibia (Boutroy et al., 2005), and to our knowledge, it has not yet been applied in FOP patients. Therefore, the aim of this case study was to assess mature HO with HR-pQCT and to compare with neighboring skeletal bone. To evaluate whether the skeletal bone of FOP patients is representative, the FOP skeletal bone and HO were also compared with an age- and gender-matched reference. It was hypothesized that mature HO contains less and thinner trabeculae and a thinner cortex compared to neighboring skeletal bone because it is often non-functional and non-weight bearing. The skeletal bone was expected to show differences with an age- and gender-matched reference because of reduced mobility related to the disease.

## Materials and Methods

### The Patients

Two FOP patients with the classical mutation (R206H), treated at the FOP Expertise Center of Amsterdam, underwent HR-pQCT imaging for this case study. Patient 1 is a 33-year-old woman who is ambulant: she can cover short distances, but she is not able to walk longer distances and to run. She is known with peripheral HO around both first metatarsals and in the left Achilles tendon. The peripheral HO has been present for over 20 years, and the patient has not noticed any flare-up or changes of this HO in the past 5 years. She has not been taking any glucocorticoids in the past 12 months but is on chronic non-steroidal anti-inflammatory drug (NSAID) treatment because of chronic pain due to HO at several sites. Patient 2 is a 23-year-old man. Peripheral HO is located around both first metatarsals and both ankles, all formed after a surgical correction of the hallux valgus early in childhood. The HO has been present for over 15 years, and the patient has not noticed any flare-up or changes of this HO in the past 5 years. He is wheelchair dependent when covering longer distances. He has not been using glucocorticoids and NSAIDs in the past 12 months. The peripheral HO in the patients was identified by physical examination and images acquired earlier by computed tomography (CT) and [^18^F] sodium fluoride (NaF) positron emission tomography (PET)-CT. They resembled mature bone as defined by a density >200 HU on CT and were not metabolically active as assessed by peak standardized uptake values on [^18^F]NaF PET-CT (Botman et al., 2019). Both patients have been participating in the LUMINA-1 clinical trial with Activin A blocking antibody Garetosmab for 3 and 4 months, respectively, at the time of HR-pQCT scanning. The HR-pQCT scans were not obtained as part of this double-blind placebo-controlled study. The patients signed the informed consent form to publish their data anonymously, and this form was approved by the Medical Ethics Review Committee of the Amsterdam UMC (Amsterdam, Netherlands).

### HR-pQCT Imaging Protocol

If the mobility of the patients allowed proper and comfortable positioning, HR-pQCT scans were obtained using the second-generation HR-pQCT scanner (XtremeCT II, Scanco Medical, Switzerland) with standard clinical settings defined by the manufacturer (X-ray tube voltage of 68 kV, intensity of 1,460 mA, and integration time of 43 ms). For the distal radius and tibia, one 10.2-mm stack was scanned at the standard location according to the standard protocol, starting 9.5 and 22.5 mm proximally from the radial and tibial endplate, respectively, and extending proximally. For the peripherally located HO, customized 30.6-mm regions (three consecutive stacks of 10.2 mm each) were scanned to ensure full capturing of the HO. The scout view, as part of the standard HR-pQCT procedure, was used to confirm a full capturing. To scan the HO in the halluces and ankles, the patients were carefully positioned with the hip and knee in flexion and the foot in plantar flexion. During acquisition of all scans, the lower arm or lower leg was placed in a standard motion restraining holder, and foam was added to the holder when necessary to ensure the patient’s comfort. Quality of the scans was graded by the operator during scan acquisition by inspection of a single low-resolution slice of each stack using the clinically used grading system provided by the manufacturer (Pialat et al., 2012). A scan was repeated when the quality of at least one stack had a grade >3 out of 5. Acquisition of one stack takes 2 min, resulting in total acquisition time of 2 min for each radius and tibia scan and 6 min for each HO scan. Effective radiation dose is approximately 5 μSv per stack, leading to an effective dose of approximately 5 μSv per radius and tibia scan and of approximately 15 μSv per HO scan. The scans were reconstructed with an isotropic voxel size of 61 μm, resulting in 168 consecutive slices per radius and tibia scan and in 504 consecutive slices per HO scan.

### Evaluation of the HR-pQCT Scans

The peripherally located HO were visually assessed by a musculoskeletal radiologist (BT) affiliated to the FOP Expertise Center; the isolated HO in the Achilles tendon of patient 1 was also quantitatively evaluated as were the distal radius and tibia. For the quantitative analysis, the isolated HO was manually segmented, and the distal radius and tibia were segmented using an automatic contouring algorithm provided by the manufacturer of the scanner. Thereafter, standard methods were used to quantify bone geometry, density, and microarchitecture of the segmented HO and distal radius and tibia. Geometric parameters included total, trabecular, and cortical area [Tt.Ar, Tb.Ar, and Ct.Ar, respectively (mm^2^)]. Densitometric parameters included volumetric bone mineral density of the entire, trabecular, and cortical bone [Tt.BMD, Tb.BMD, and Ct.BMD, respectively (mg HA/cm^3^)]. Microarchitectural parameters included trabecular bone volume fraction [Tb.BV/TV (−)], trabecular number [Tb.N (mm^–1^)], thickness [Tb.Th (mm)], separation [Tb.Sp (mm)], and heterogeneity [Tb.1/N.SD (mm)], and cortical thickness [Ct.Th (mm)], and porosity [Ct.Po (−)]. Additionally, failure load (FL) was estimated of the segmented HO and distal radii and tibiae by means of micro-finite element (μFE-) modeling. Linear three-dimensional μFE-models were generated by converting the bone voxels of the HR-pQCT scans to equally sized brick elements, which were assigned a Poisson’s ratio of 0.3 and a Young’s modulus of 8,748 MPa (Whittier et al., 2020). An axial compression to 1% strain was then simulated along the longitudinal axis (*i.e.*, compression with constraint of lateral expansion at the bone endings) to estimate FL, for which Pistoia’s criterion was used (Pistoia et al., 2002). For this estimation of FL, it was assumed that the HO experiences a tensile load in the direction of the calve muscles and thus in the direction of the compression load on the tibia; simulating either tension or compression gives, apart from the sign, the same results in FE-modeling.

The resulting values of the bone parameters from all analyses were compared to an age- and gender-matched normative reference group. This normative dataset was obtained in a general Canadian male and female population using the same generation HR-pQCT scanner and analyses as in this case study and has recently been published by Whittier et al. (2020).

## Results

### Scan Acquisition

The patients’ mobility allowed acquisition of HR-pQCT scans of HO in the left Achilles tendon and around both metatarsals of patient 1 and around the right ankle of patient 2. The HO in the Achilles tendon was visible on the standard scan of the left tibia and required no separate scan. HR-pQCT scans could not be acquired of the halluces and left ankle of patient 2 as he was not able to position his foot in plantar flexion due to ankle ankylosis. His right ankle could be scanned without plantar flexion of the foot, but with additional foam for comfort during scan acquisition. Due to the inability of both patients to properly position before the scanner, a distal radius scan could only be made of the left side of patient 1. Distal tibia scans could be obtained of the left and right tibia of both patients. All obtained HR-pQCT scans were of good quality (≤grade 3 for all stacks); therefore, none of the scans had to be repeated.

### Evaluation of the Distal Radius and Tibia

**FIGURE 1:**
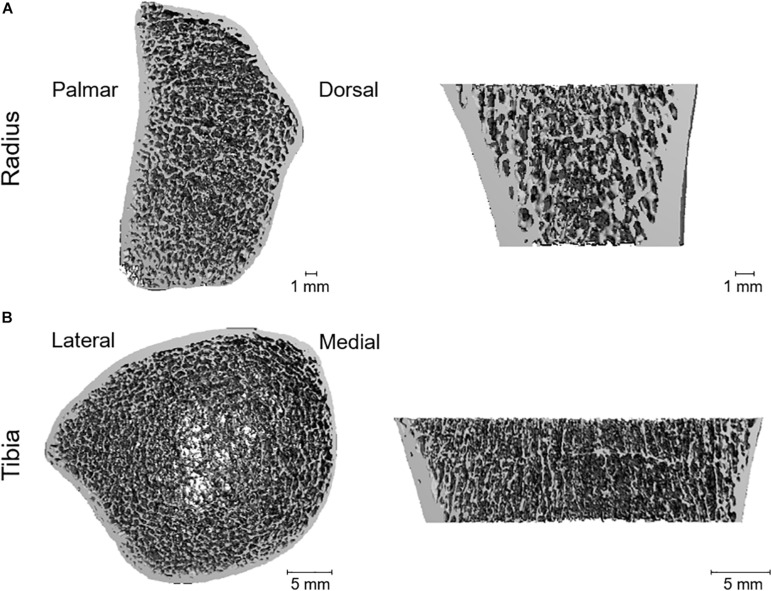
Three-dimensional visualization of the scanned left radius **(A)** and right tibia **(B)** of patient 1, obtained with HR-pQCT. **(A)** Quantification revealed a great resemblance of the radius of the fibrodysplasia ossificans progressiva (FOP) patient to that of an age- and gender-matched reference group, except for cortical density that was higher in the FOP patient. **(B)** Quantification revealed major dissimilarities with the age- and gender-matched reference group, especially in total and trabecular density (lower in the patient) and in trabecular and cortical microarchitecture.

A three-dimensional visualization of the scanned left radius and right tibia of patient 1 is shown in Figure 1. Geometry, density, microarchitecture, and FL of the dominant side, the left radius of this patient, were within the 25^th^–75^th^ percentile (pctl), except for Ct.BMD that was considerably higher (90^th^–98^th^ pctl) (Table 1). At the tibia, both patients had low Tt.BMD and Tb.BMD compared to the age- and gender-matched reference group (<2^nd^ pctl) (Tables 1, 2). The trabecular microarchitectural parameters were below or above the interquartile range (IQR): Tb.BV/TV, Tb.N, and Tb.Th were lower (<2^nd^ pctl, < 25^th^ pctl, and <2^nd^ pctl, respectively), and Tb.Sp and Tb.1/N.SD were higher (>75^th^ pctl and >90^th^ pctl, respectively). The FL of the tibiae of both patients was also lower than normative values (<2^nd^ pctl). In patient 1, Ct.Ar and Ct.Th were lower in both tibiae (both <10^th^ pctl), and Ct.BMD was in the lower half of the IQR. In patient 2, Ct.Ar and Ct.Th were <25^th^ pctl in the right tibia, and Ct.BMD was in the upper half of the IQR in both tibiae.

**TABLE 1:** Bone parameters of the left distal radius and left and right distal tibia of patient 1, a 33-year-old woman including normative values of the distal radius and tibia of age- and gender-matched controls.

	Radius				Tibia			
	Left Value	Percentile^a^	Right Value	Percentile^a^	Left Value	Percentile^a^	Right Value	Percentile^a^
**Geometry (area; mm^2^)**								
Tt.Ar	247.7	25–75	–	–	714.6	75–90	709.1	25–75
Tb.Ar	192.9	25–75	–	–	627.5	75–90	622.8	75–90
Ct.Ar	58.2	25–75	–	–	92.6	2–10	91.6	2–10
**Density (BMD; mg HA/cm^3^)**								
Tt.BMD	336.0	25–75	–	–	205.3	<2	202.6	<2
Tb.BMD	143.1	25–75	–	–	100.9	<2	95.2	<2
Ct.BMD	991.2	90–98	–	–	934.8	25–75	955.5	25–75
**Microarchitecture**								
Tb.BV/TV (−)	0.187	25–75	–	–	0.143	<2	0.127	<2
Tb.N (mm^–1^)	1.442	25–75	–	–	1.149	10–25	1.162	10–25
Tb.Th (mm)	0.211	25–75	–	–	0.209	<2	0.202	<2
Tb.Sp (mm)	0.649	25–75	–	–	0.858	75–90	0.855	75–90
Tb.1/N.SD (mm)	0.225	25–75	–	–	0.420	90–98	0.380	90–98
Ct.Th (mm)	1.043	25–75	–	–	1.078	2–10	1.013	<2
Ct.Po (−)	0.002	25–75	–	–	0.008	25–75	0.009	25–75
**Mechanics (μFE)**								
FL (kN)	2.68	25–75	–	–	5.28	<2	4.88	<2

*Tt: total: Tb: trabecular; Ct: cortical; Ar: area; BMD: volumetric bone mineral density; Tb.BV/TV: trabecular bone volume fraction; Tb.N: trabecular number; Tb.Th: trabecular thickness; Tb.Sp: trabecular separation; Tb.1/N.SD: heterogeneity of the trabecular network; Ct.Th: cortical thickness; Ct.Po: cortical porosity; FL: estimated failure load. ^*a*^Percentile is based on comparison with an age- and gender-matched reference from literature (Whittier et al., 2020).*

**TABLE 2:** Bone parameters of the left and right distal tibia of patient 2, a 23-year-old man including normative values of the distal radius of age- and gender-matched controls.

Tibia	Left Value	Percentile^a^	Right Value	Percentile^a^
**Geometry (area; mm^2^)**				
Tt.Ar	849.5	25–75	831.3	25–75
Tb.Ar	718.9	25–75	713.7	25–75
Ct.Ar	136.4	25–75	123.3	2–10
**Density (BMD; mg HA/cm^3^)**				
Tt.BMD	229.1	<2	232.8	<2
Tb.BMD	98.4	<2	113.9	<2
Ct.BMD	932.5	25–75	936.9	75–90
**Microarchitecture**				
Tb.BV/TV (−)	0.1558	<2	0.172	<2
Tb.N (mm^–1^)	0.855	<2	1.086	2–10
Tb.Th (mm)	0.235	<2	0.233	<2
Tb.Sp (mm)	1.170	>98	0.902	90–98
Tb.1/N.SD (mm)	0.827	>98	0.403	90–98
Ct.Th (mm)	1.510	25–75	1.318	10–25
Ct.Po (−)	0.012	25–75	0.007	10–25
**Mechanics (μFE)**				
FL (kN)	8.21	<2	7.82	<2

*Tt: total; Tb: trabecular; Ct: cortical; Ar: area; BMD: volumetric bone mineral density; Tb.BV/TV: trabecular bone volume fraction; Tb.N: trabecular number; Tb.Th: trabecular thickness; Tb.Sp: trabecular separation; Tb.1/N.SD: heterogeneity of the trabecular network; Ct.Th: cortical thickness; Ct.Po: cortical porosity; FL: estimated failure load. ^*a*^Percentile is based on comparison with an age- and gender-matched reference from literature (Whittier et al., 2020).*

### Evaluation of Heterotopic Ossification

**FIGURE 2:**
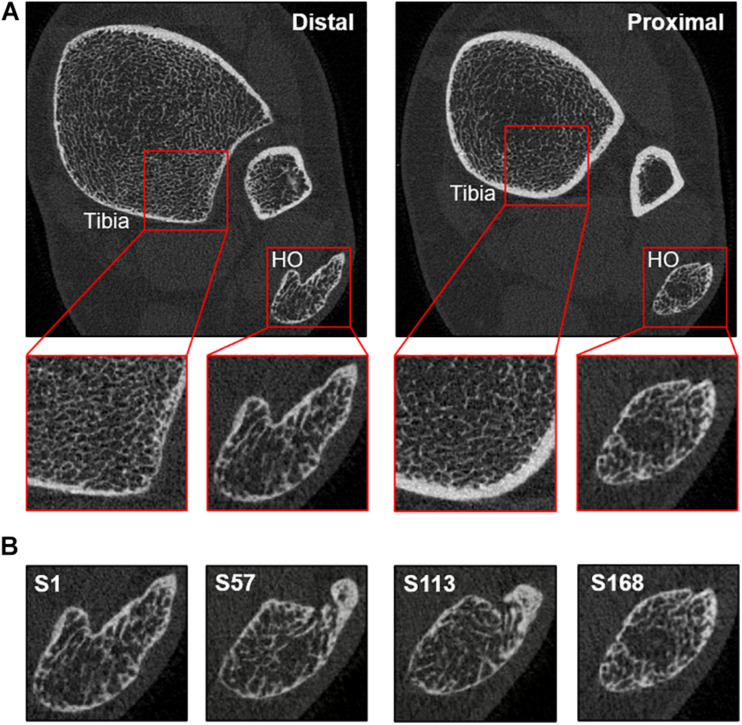
**(A)** Distal (left) and proximal (right) slice of the HR-pQCT scan of the left tibia of patient 1, a 33-year-old female fibrodysplasia ossificans progressiva (FOP) patient showing isolated heterotopic ossification (HO) in the Achilles tendon. Quantification showed dissimilarities between the HO and the patient’s tibia, especially in trabecular microarchitecture when compared to an age- and gender-matched reference. **(B)** Four slices from distal (left) to proximal (right) of the HR-pQCT scan of the left tibia showing HO in the Achilles tendon.

The HO in the left Achilles tendon of patient 1 was isolated from the neighboring skeletal bone in the scanned region and could therefore be qualitatively as well as quantitatively evaluated (Figure 2). The HO constituted a clear cortex and trabecular structure. The cortex appeared thinner than in the neighboring left tibia, but a thickening was present in the axial middle of the scanned region of the HO. The results of the quantitative analysis of the isolated HO and neighboring left tibia are presented in Table 3. In the isolated HO, Ct.BMD was below the IQR of the normative dataset (< 2^nd^ pctl), while it was within the IQR in the left distal tibia. Tt.BMD, Tb.BMD, and Ct.Th were below the IQR for both the isolated HO and left tibia. The trabecular microarchitectural parameters were within the IQR for the HO except for Tb.BV/TV (10^th^–25^th^ pctl), whereas for the left tibia, Tb.BV/TV, Tb.N, and Tb.Th were lower than the IQR (<2^nd^ pctl, 10^th^–25^th^ pctl, and <2^nd^ pctl, respectively), and Tb.Sp and Tb.1/N.SD were higher (75^th^–90^th^ pctl and 90^th^–98^th^ pctl, respectively). FL was approximately a factor 10 lower in the HO than in the left tibia. It was low compared to normative values (<2^nd^ pctl). Total bone area was approximately a factor 10 higher in the HO than in the tibia.

**TABLE 3:** Bone parameters of the left distal tibia and isolated heterotopic ossification (HO) in the left Achilles tendon of patient 1, a 33-year old woman including normative values of the distal tibia with age- and gender-matched controls.

	HO (left Achilles tendon)		Left tibia	
	Value	Percentile^a^	Value	Percentile^a^
**Geometry (area; mm^2^)**				
Tt.Ar	64.9	<2	714.6	75–90
Tb.Ar	49.6	<2	627.5	75–90
Ct.Ar	17.2	<2	92.6	2–10
**Density (BMD; mg HA/cm^3^)**				
Tt.BMD	271.9	10–25	205.3	<2
Tb.BMD	142.1	10–25	100.9	<2
Ct.BMD	656.7	<2	934.8	25–75
**Microarchitecture**				
Tb.BV/TV (−)	0.195	10–25	0.143	<2
Tb.N (mm^–1^)	1.242	25–75	1.149	10–25
Tb.Th (mm)	0.254	25–75	0.209	<2
Tb.Sp (mm)	0.766	25–75	0.858	75–90
Tb.1/N.SD (mm)	0.282	25–75	0.420	90–98
Ct.Th (mm)	0.917	<2	1.078	2–10
Ct.Po (−)	0.013	25–75	0.008	25–75
**Mechanics (μFE)**				
FL (kN)	0.530	<2	5.28	<2

*Tt: total; Tb: trabecular; Ct: cortical; Ar: area; BMD: volumetric bone mineral density; Tb.BV/TV: trabecular bone volume fraction; Tb.N: trabecular number; Tb.Th: trabecular thickness; Tb.Sp: trabecular separation; Tb.1/N.SD: heterogeneity of the trabecular network; Ct.Th: cortical thickness; Ct.Po: cortical porosity; FL: estimated failure load. ^*a*^Percentile is based on comparison with an age- and gender-matched reference from literature (Whittier et al., 2020).*

**FIGURE 3:**
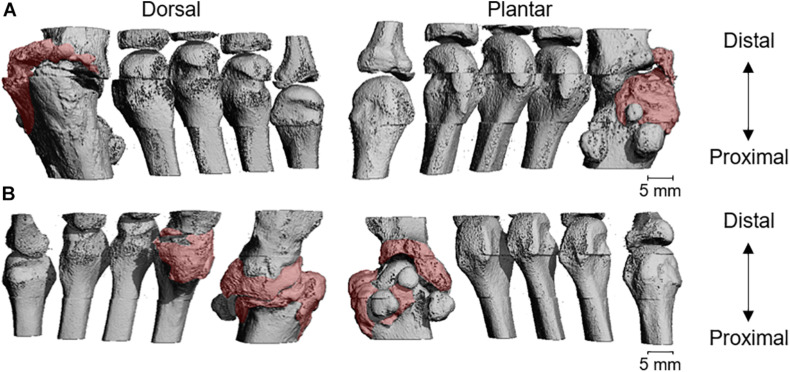
Three-dimensional visualization of the scanned metatarsals of the right **(A)** and left **(B)** foot of patient 1. The patient underwent surgery for the correction of a bilateral hallux valgus at the age of 1, resulting in heterotopic ossification (HO) at the operated sites. The pink color visualizes the sites with HO. It should be noted that, due to fusion, the exact border between HO and skeletal bone could not be established.

**FIGURE 4:**
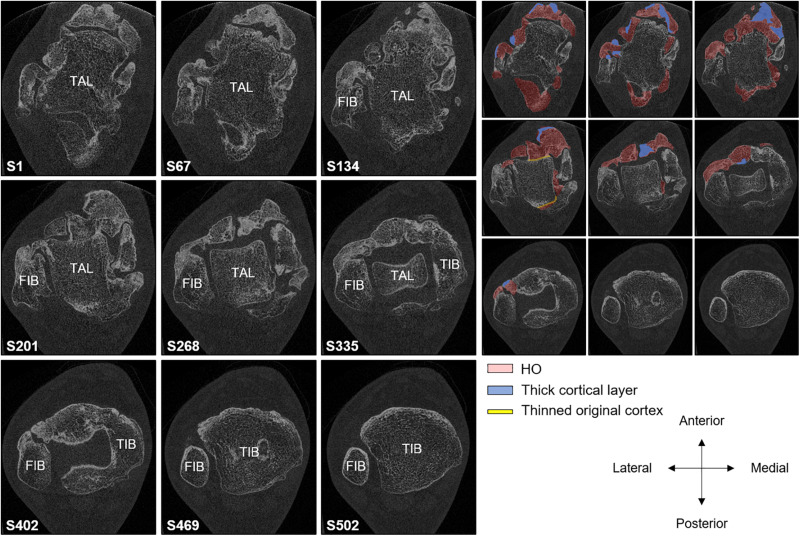
Nine two-dimensional slices of the HR-pQCT scan of a 30.6-mm region of the right ankle of patient 2, a 23-year-old male fibrodysplasia ossificans progressiva (FOP) patient, from distal (S1) to proximal (S502). S1 shows the talus with heterotopic ossification (HO) attached. The original cortical layer of the talus seems disrupted at several sites where HO has fused. (S67–S502) HO has also fused to the fibula. However, a thin cortical layer seems to be present throughout the series. (S201–S502) HO has fused to the tibia; as with the talus, the cortical layer is disrupted at several sites of the fusion. TIB, TAL, and FIB represent tibia, talus, and fibula, respectively. On the right, HO, increased cortical bone, and the thinned cortex are visualized in blue, red, and yellow, respectively. It should be noted that, due to fusion, the exact border between HO and skeletal bone could not be established.

The HR-pQCT scans obtained of the other peripheral HO in both patients revealed fusion with the neighboring skeletal bone and could only be qualitatively analyzed. Figure 3 shows a three-dimensional image of the halluces of patient 1. As can be seen, HO was fused with the phalanx, and at several sites where HO had adjoined the phalanx, the initial cortex was replaced by trabeculae. The trabecular compartments of the HO and phalanx seemed to merge and were surrounded by a cortex of the HO. This neo-cortex appeared thinner than the initial cortex of the phalanx, whereas the trabeculae appeared thicker (data not shown). A similar pattern was found in the right ankle of patient 2 (Figure 4): there was fusion of HO with the tibia, fibula, and talus, with partial replacement of the original cortex by trabeculae and a (neo-)cortex surrounding the fused bone structures. Enlargement of the distal tibia was also observed. The HO adjoining the ankle bones seemed to contain relatively more cortical bone than the ankle bones and the other HO lesions inspected.

Although all HO lesions analyzed by HR-pQCT had a density of >200 HU and thus resembled mature bone, HU was lower in these lesions than in neighboring skeletal bone that had a density of >350 HU. The only exception was the HO around the ankle, which had a considerable higher HU value than the surrounding talus, tibia, and fibula.

## Discussion

The aim of this case study was to assess peripherally located mature HO and skeletal bone of two FOP patients with HR-pQCT and to compare those with each other and with an age- and gender-matched reference group from literature. The HO assessed in both patients was found to merge with neighboring skeletal bone. The cortex of the skeletal bone at sites of fusion appeared to be replaced by trabeculae to form one new entity, constituting trabecular bone surrounded by a (neo-)cortex. Most bone parameters of the isolated HO in the Achilles tendon of one of the patients were found to be within the IQR of age- and gender-matched reference tibia data, whereas most of the neighboring tibia were below or above the IQR. The bone parameters of the distal radius resembled the literature values, except for cortical BMD.

To the best of our knowledge, this is the first study examining HO non-invasively and at a microarchitectural level in living FOP patients. HO around the halluces and ankle of the patients was merged with the neighboring skeletal bone, and it appeared that a neo-cortex was formed, surrounding the HO where it fused with the skeletal bone. However, the thin lining of the original cortical layer of the skeleton was still visible at various regions, suggesting a yet incomplete coalescence. This may indicate that the fusion of HO and skeletal bone and associated remodeling is a slow process, considering the presence of the HO for over 15 years in both patients. The neo-cortex around the halluces appeared thinner than the original skeletal cortex, whereas HO around the ankle consisted of a relatively thick cortical layer, which is perhaps due to the weight-bearing function of the ankle and its associated HO. Both these fused HO and the isolated HO assessed in this case study showed a cortical and trabecular compartment as do skeletal bones, which, in that respect, agrees with previous case reports suggesting that HO of FOP patients has a similar morphology as the skeletal bone (Kaplan et al., 1993; Mahboubi et al., 2001). However, these earlier publications have mainly investigated biopsies of early HO lesions that were taken in children for other reasons (Kaplan et al., 1993; Jayasundara et al., 2012; Kamal et al., 2015), while we investigated mature HO lesions as confirmed by CT. Furthermore, the use of HR-pQCT instead of histology enables a quantitative evaluation of HO besides a qualitative assessment, which may provide new insights into HO in FOP.

Today, quantitative assessment of the microarchitecture of HO is scarce. Most mice studies mimicking FOP and HO formation have not quantified the HO microstructure despite the use of high-resolution imaging modalities (*e.g*., μCT) (Brownley et al., 2015; Hatsell et al., 2015; Chakkalakal et al., 2016; Upadhyay et al., 2017). In humans, the only study quantifying HO at the microarchitectural level concerned, to our knowledge, an *ex vivo* μCT study on bone biopsies of non-genetic HO. In that study, surgically removed HO at muscular tissue was analyzed, which revealed variations in microarchitecture between and within lesions and an affected strength of the HO lesions compared to the skeletal bone (Trieb et al., 2018). In contrast, we found FL of the isolated HO in the Achilles tendon to be approximately 10% higher than of the neighboring tibia when correcting for the difference in total bone area. This agrees with the assumption in literature that the strength of HO in FOP is preserved, which is based on the observation that (stress) fractures of HO are not often seen in FOP patients (Einhorn and Kaplan, 1994). The discrepancy in the findings on strength between the study on non-genetic HO and our case study on genetic HO may, among others, be caused by a different ossification process with distinct histological characteristics between genetic and non-genetic HO (Meyers et al., 2019). Furthermore, HO in tendons may not be representative for HO in muscles as, for example, trauma-induced muscle HO in FOP appears to be driven through another progenitor lineage than HO formed in ligaments and tendons (Dey et al., 2016). This may also contribute to the qualitative differences found at the microarchitectural level between the trauma-induced HO around the ankle of patient 2 and the spontaneously formed HO in the Achilles tendon of patient 1. Notably, the estimated FL in the isolated HO in the Achilles tendon is lower than the peak forces that occur in the Achilles tendon during daily life in healthy individuals [*e.g.*, 1.3–1.5 kN during walking, up to 4 kN during running and jumping (Fukashiro et al., 1995; Finni et al., 1998; Hosseini et al., 2017)], which would suggest rupture of the Achilles tendon or fracture of the HO during such activities. However, such peak forces may not be reached in FOP patients due to their reduced ability or inability to fully perform these activities.

The comparison of the quantitative evaluation of the HO in the Achilles tendon with the neighboring tibia showed that the microarchitectural parameters of the HO had better agreement with the age- and gender-matched reference than the left tibia. It is not known what may have caused this difference between HO and neighboring tibia. A different mechanical stimulation may possibly play a role, but a study in larger datasets is needed before any conclusions can be drawn about the possible microarchitectural differences between HO and skeleton. These microarchitectural differences could have contributed to the 10% larger estimated FL of the HO compared to the tibia after accounting for areal differences. The comparison of the HO with the peripheral skeleton raises the question on whether the skeletal bone of FOP patients is comparable at the microarchitectural level to an age- and gender-matched reference (Burt et al., 2016). Unlike microarchitectural misbalances throughout the entire skeleton in other rare bone diseases, the microarchitecture of the analyzed radius of patient 1 was found to be comparable to an age- and gender-matched reference population (Folkestad et al., 2012; Kocijan et al., 2015; Arruda et al., 2016; Butscheidt et al., 2018). The tibiae of both FOP patients, in contrast, did show considerable deviations from age- and gender-matched normative data: the total and trabecular BMD were lower, and the trabecular compartment consisted of less and thinner trabeculae in a more heterogeneously formed network. Reduced mobility or a changed mechanical loading in the FOP patients could have contributed to these differences from the normative data, as could have the frequent glucocorticoid use of both patients (Ilias et al., 2000). Research in larger datasets is needed to further investigate possible microarchitectural differences between the skeleton of FOP patients and of the general population.

This study has shown that HR-pQCT allows visualization and quantification of BMD, microarchitecture, and the biomechanical properties of mature HO in FOP patients *in vivo*. This imaging modality enables analysis of mature HO in more detail than other imaging modalities while simultaneously exposing patients to a negligible amount of radiation. Furthermore, it is non-invasive, in contrast to bone biopsy for histological analysis; therefore, it does not trigger flare-ups as is the case with biopsy. Consequently, HR-pQCT may be an interesting technique for future research into mature HO in FOP, such as for sequential HR-pQCT imaging to study the development of mineralized HO during a flare-up or the effects of a study drug on mature HO. However, it is likely that the ability of FOP patients to properly position before the gantry of the scanner is restricted as shoulders, elbows, hips, and knees are frequently ankylosed in these patients (Pignolo et al., 2016), which may limit scan acquisition and affect scan quality. The right distal radius of patient 1 and both distal radii and the right ankle of patient 2 could not be scanned for that reason. As a result, it may be challenging to find patients eligible for HR-pQCT scanning, combined with the rarity of FOP and the restriction to peripheral bones, as HO in FOP patients mainly forms more centrally, while peripheral sites are often spared (Smith et al., 1976; Rogers and Geho, 1979; Connor and Evans, 1982; Pignolo et al., 2016).

Several limitations of this case study have to be mentioned. First, we examined HO of two FOP patients, which gives an interesting impression about the skeletal microarchitecture of bone in FOP patients but does limit interpretation. Larger datasets are needed to draw conclusions on the possible differences in BMD, microarchitecture, and strength between HO and skeletal bone and between the skeletal bone of FOP patients and an age- and gender-matched reference population. Second, the estimation of FL using μFE-analysis and Pistoia’s criterion is only validated for the distal radius, and thus its accuracy is not known for HO, further limiting the conclusions on the strength of HO compared to that of the skeletal bone. Third, the two patients in this study were participating in a double-blind placebo-controlled trial at the time of HR-pQCT imaging, and it is unknown whether they were on active treatment that could have influenced the bone parameters. Finally, the quantitative analysis of HO was limited to HO in the Achilles tendon of patient 1 as the other scanned HO was not isolated from the neighboring skeletal bone, which made it impossible to establish the exact border between the HO and the skeletal bone for segmentation of the HO. Nevertheless, qualitative analysis was possible for this HO.

In conclusion, this case study showed that HR-pQCT allows a non-invasive assessment of peripherally located HO and distal radius and tibia in FOP patients, which may provide new insights into skeletal and heterotopic bone in FOP patients at the microarchitectural level. Isolated HO seemed microarchitecturally more comparable to skeletal bone from reference data than the peripheral skeleton of FOP patients. Additionally, HO and skeleton appear to become one entity when contiguous.

## Data Availability Statement

The datasets presented in this article are not readily available because the data remains in the posession of the Amsterdam UMC. Requests to access the datasets should be directed to EB, e.botman@amsterdamumc.nl.

## Ethics Statement

The studies involving human participants were reviewed and approved by Medische Ethische Toetsingscommissie (METC) Amsterdam UMC, locatie VUmc, Netherlands. The patients/participants provided their written informed consent to participate in this study.

## Author Contributions

EB, CW, JN, JB, and EE contributed to the conception, methodology, and investigation of the study. MB validated the data, performed the analysis, and contributed to the visualization of the data. EB and MB wrote the original draft and reviewed and edited the manuscript. BR contributed to the validation of data. BR, BT, and PR contributed to the formal analysis. CW, BR, BT, PR, JN, JB, and EE reviewed the manuscript. All the authors contributed to manuscript revision, read, and approved the submitted version.

## Conflict of Interest

BR is an external consultant for Scanco Medical. The remaining authors declare that the research was conducted in the absence of any commercial or financial relationships that could be construed as a potential conflict of interest.
